# Addressing Key Clinical Care and Clinical Research Needs in Severe Pediatric Traumatic Brain Injury: Perspectives From a Focused International Conference

**DOI:** 10.3389/fped.2020.594425

**Published:** 2021-01-18

**Authors:** Mirco Nacoti, Francesco Fazzi, Francesco Biroli, Rosalia Zangari, Tiziano Barbui, Patrick M. Kochanek, The Collaborative Pediatric TBI Working Group, Guido Bertolini

**Affiliations:** ^1^Pediatric Intensive Care Unit, Department of Anesthesia and Intensive Care, Papa Giovanni XXIII Hospital, Bergamo, Italy; ^2^Fondazione per la Ricerca dell'Ospedale di Bergamo Research Foundation, Papa Giovanni XXIII Hospital, Bergamo, Italy; ^3^Department of Critical Care Medicine, Safar Center for Resuscitation Research, John G Rangos Research Center, University of Pittsburgh Medical Center Children's Hospital of Pittsburgh, University of Pittsburgh School of Medicine, Pittsburgh, PA, United States

**Keywords:** traumatic brain injury, pediatric, intracranial pressure, outcomes, prognosis

## Abstract

Traumatic brain injury (TBI) is a leading cause of morbidity and mortality in children and adolescents. Survivors of severe TBI are more prone to functional deficits, resulting in poorer school performance, poor health-related quality of life (HRQoL), and increased risk of mental health problems. Critical gaps in knowledge of pathophysiological differences between children and adults concerning TBI outcomes, the paucity of pediatric trials and prognostic models and the uncertain extrapolation of adult data to pediatrics pose significant challenges and demand global efforts. Here, we explore the clinical and research unmet needs focusing on severe pediatric TBI to identify best practices in pathways of care and optimize both inpatient and outpatient management of children following TBI.

## Introduction

Traumatic brain injury (TBI) represents a global health problem of substantial proportions. Worldwide, it is the leading cause of mortality in young adults and a major cause of injury-related death and disability across all ages in the majority of countries (1). Recent estimates of the global, regional and national burden in terms of incidence and years of life lived with disability reveal a disproportionate TBI burden in low–middle-income countries (LMICs) vs. high-income countries (HICs) (1, 2). Despite this, LMICs are often poorly resourced to conduct trials and publish data on TBI vs. HICs (3). Addressing this disparity is crucial to implement optimal global prevention strategies, expand current knowledge on TBI patterns and to develop practical clinical care recommendations with a worldwide perspective (4).

Age is among the strongest outcome predictors of outcome in TBI with children and elderly being the most vulnerable populations (1, 5). The functional impact of TBI in children goes far beyond the acute injury. While some cognitive, motor, and behavioral sequelae are immediately apparent, others may emerge later when a child re-attends school or an infant fails to develop as expected. Furthermore, pediatric TBI (pTBI) care suffers from a number of other limitations, including (1) a long-standing underestimation of the anatomical and physiological differences, relevant to TBI, between children and adults; (2) inappropriate extrapolation of adult data to pediatrics that often occurs due to the limited clinical evidence on which recommendations are based; and (3) scarce pre-clinical and clinical research specifically focused on pTBI (5). As a result, children are at high risk to receive suboptimal care (6). These challenges in pTBI, encompassing systems of care, clinical management and research strategy, demand novel approaches to produce new evidence to be implemented into clinical practice (7). In this regard, Comparative Effectiveness Research (CER) studies, such as CENTER-TBI (in adult and pediatric patients) and ADAPT (Approaches and Decisions in Acute Pediatric TBI) trial, are promising novel approaches that may help to fill the gap between adult and children knowledge and between HICs and LMICs outcome (8).

This work integrates the key insights stemming from an international conference and explores the clinical care and research priority issues to inform and stimulate further initiatives aimed at ensuring an adequate and effective pediatric-tailored TBI management.

Selection of evidence:

Papers for consideration for the present narrative review were identified by a PubMed search, using different combinations of pertinent keywords (e.g., traumatic brain injury AND management). Only articles published in English during the last 20 years were included. Papers were selected for inclusion according to their relevance for the topic, as judged by the Authors.

## From Epidemiology to Patient Management: Current Knowledge and Gaps in Pediatric TBI

### TBI Burden Estimation: the Need for Standardized Epidemiological Monitoring

pTBI is the most common cause of death in children and young adults worldwide with a high variability in incidence across diverse geographic regions with most reporting a range between 47 and 280 per 100,000 children (9, 10).

Reliable quantification of the burden of TBI is difficult to achieve due to inadequate standardization of data definitions and severity classification. Common data elements have only recently begun to be standardized and harmonized in pediatric TBI (11). Approximately 80% of pTBI cases can be classified as mild (Glasgow Coma Scale [GCS] score ≥13) with negative imaging findings and these cases are often seen by family practitioners; thus, epidemiological estimation based on hospital admission or emergency department (ED) visits underestimates the true TBI incidence as much as 4–5-fold (12). Similarly, in LMICs, access to healthcare resources may be challenging and result in a falsely low prevalence of TBI diagnosis, including children who die before hospitalization (1). Despite the magnitude of this burden, data to characterize the etiology and risk factors associated with childhood injuries are limited, especially in LMICs. Overall, accurate measurement of incidence, prevalence, morbidity, mortality, and rates of access to community, hospital and rehabilitative, and outpatient care across the globe are urgently needed as they can inform public prevention measures and appropriate allocation of healthcare resources and research priorities.

Summary of gaps and actionable suggestions

- Reliable quantification of the burden caused by TBI is difficult owing to inadequate standardization of data entry and a substantial variability in TBI definition and severity classification
- Centralization of epidemiological data in local and national registries, as well a better integration between existing trauma registries may contribute to an improved pTBI burden estimation (useful also in LMICs)
- Development of context-specific clinical practice guidelines and building global research collaborations to bridge the existing gaps in knowledge on all aspects of the provision of care (useful also in LMICs).

### Pre-Hospital Care

Patients with TBI, particularly those with moderate or severe injury, need timely-efficient and specialized care. Thus, pre-hospital care initiates the chain of trauma care that includes a spectrum of caregivers and skills: first responders, dispatch systems, basic life support, mobile medical teams, helicopter emergency medical services (HEMS), and hospital choice (1). There is a long-standing controversy on the optimal approach—that is, whether it is beneficial to stabilize patients on the scene of injury before the transfer or to transfer them to hospital as rapidly as possible (stay-and-play vs. scoop-and-run approaches) (1).

Helicopter use and its impact on TBI outcomes remain controversial (1, 13). Several pre-hospital major trauma patient triage scores have been developed and are currently used, (i.e., the triage revised trauma score -T-RTS, pediatric trauma score- PTS, Vittel criteria, Mechanism/GCS/Age/Systolic blood pressure score -MGAP, the new trauma score –NTS, and the National Advisory Committee for Aeronautics severity score -NACA-SS) but none of them has demonstrated a clear superiority over the others. Available evidence on air vs. ground transport mostly explores advantages of helicopter use in terms of time effectiveness while raising concern on its economic sustainability (14). Scarce and not always comparable data are also available about the overall clinical impact of mode of transport (15). Thus, future research should aim at addressing the appropriateness of helicopter transport in terms of clinical outcome and interventions upon hospital arrival, rather than being focused solely on time-saving. Furthermore, an effort must be made to validate tools that allow adequate patient triage. In addition, from a pathophysiological perspective, it has been postulated that helicopter transport may impair brain perfusion in patients suffering from TBI. After initiating intracranial pressure (ICP)-lowering strategies on the scene, helicopter transportation may counteract their benefit by the use of the in-flight Trendelenburg position which produces cerebral venous blood pooling (16). The effects of helicopter transport on secondary neurological damage have yet to be evaluated in-depth; nevertheless, in moderate/severe injuries, HEMS seems to be associated with decreased mortality, potentially saving one life for every 47 flights (17). Critically injured patients, who potentially have the most to gain by rapid transport to definitive care, may not all be optimally served by air transfer given the increased risk of sustaining a secondary injury, due to uncontrolled ICP (16). Addressing both in-hospital and pre-hospital management of severe TBI is required to reduce the high mortality in LMICs. Fifty percent of patients who die from TBI do so within the first 2 h of injury (18). Patients who die before reaching a hospital in LMIC is over twice that of HICs (19). Overall, while pre-hospital care is an evolving field in many LMICs we continuelack on necessary emergency medical care services that would improve outcomes in resource-limited countries (20).

Finally, a recent study of TBI management in four resource limited setting in Africa revealed that although EMS was described as available, it was not often used and most of the patients arrived by taxi, bus, or private vehicle (21). In addition, EMS personnel were trained only in basic life support. In that report, helicopter transport was generally not available, and the report concluded that in most cases it was not required.

Summary of gaps and actionable suggestions

- Inter-hospital transfers are 2-times higher for children compared with adult patients and over-triage occurs more frequently in children compared with adult patients (89 vs. 81%).
- The overall impact of mode of transport on clinical outcomes is not yet established.
- Triage of pediatric TBI patients should be ensured to pediatric trauma centers.
- Increased efforts are needed to develop a highly sensitive and specific pediatric trauma triage tools to aid decision-making and to ensure accuracy of field triage and associated diagnostic protocols.
- Future work should consider how to categorize pediatric injuries and determine which mechanisms predict the need for referral to a trauma center.

### Pediatric Guideline Update 2019 and Algorithm: a Pragmatic Pathway to Improve Pediatric TBI Management

In late 2019, the Third Edition of the Brain Trauma Foundation (BTF) guidelines for hospital care for severe pTBI was published as three distinct documents: the full guidelines, an executive summary and a treatment algorithm. A total of 22 recommendations with nine being new or revised from previous editions were presented (22, 23). However, none are level I, three are level II and 19 are level III. A detailed description of guidelines recommendations is beyond the scope of this review and we refer the reader to the original publications (22, 23). Relevant methodological insights and clinical perspectives should be highlighted as they have mostly contributed to the revision of previous recommendations.

For the first time, ICP control was considered an outcome along with mortality. The authors, while acknowledging that any conclusive demonstration that ICP-targeted therapy would improve long-term outcome is lacking, recommend the inclusion of this outcome by virtue of the high-quality evidence stemming from studies having ICP control as primary endpoint. A high variability in locally used treatment protocols, particularly for topics with limited evidence (e.g., ICP control) has been reported by both American and European TBI centers (23). Thus, both stood as an integral part of TBI management despite the lack of evidence with all centers unanimously reporting the use of an ICP threshold of 20 mmHg (24). Nevertheless, three new class III retrospective observational studies were added to the evidence base for this topic. Apparently divergent recommendations were made on the approach to temperature control depending on the considered outcome–either ICP control or overall outcome. Prophylactic mild hypothermia—as a first-tier therapy for all severe TBI cases is not recommended, while hypothermia has support as an option for second-tier use in the setting of refractory intracranial hypertension (Table 1). This approach highlights how effects of therapies on both ICP and long-term outcome can help guide provision of care in TBI patients.

**Table 1:** Summary of the main new Brain Trauma Foundation 2019 recommendations listed by topic.

**Topic**	**Level**	**Recommendation**	**Updated content**
Neuroimaging	III	To improve overall outcome	CT examinations should not be used to rule out the possibility of elevated ICP
Hyperosmolar therapy	II	For ICP control	Hypertonic saline (3%) is recommended at doses of 2–5 ml/kg over 10–20 min
	III	For ICP control	Hypertonic saline (23.4%) is suggested for refractory ICP at doses of 0.5 ml/kg
		Safety	To avoid sustained (>72 h) serum sodium >170 mEq/l is suggested to obviate anemia and thrombocytopenia; avoiding >160 mEq/l to circumvent DVT
Sedation, analgesia and neuromuscular blockade	III	For ICP control	Avoid bolus of midazolam and/or fentanyl to control ICP because of risk of cerebral hypoperfusion
Seizure prophylaxis	III	Seizure prevention	Insufficient evidence to recommend levetiracetam over phenytoin based on efficacy either toxicity
Temperature control	II	To improve overall outcome	Prophylactic moderate hypothermia (32–33°C) is not recommended over normo-thermia
	III	For ICP control	Moderate hypothermia is suggested for ICP control
		Safety 1	Rewarming should be carried out at a rate of 0.5–1°C every 12–24 h
		Safety 2	If phenytoin is used during hypothermia monitoring level is suggested to minimize toxicity
Nutrition	III	To improve overall outcome	Early (within 72 h from injury) enteral nutrition is suggested
Corticosteroids	III	To improve overall outcome/for ICP control	The use of corticosteroids is not suggested Note: Previous recommendation is not intended to circumvent the use of replacement corticosteroids (chronic therapy, adrenal suppression, injury of the hypothalamic pituitary axis)

*CT, computed tomography; DVT, deep vein thrombosis; ICP, intracranial pressure*.

**Figure 1:**
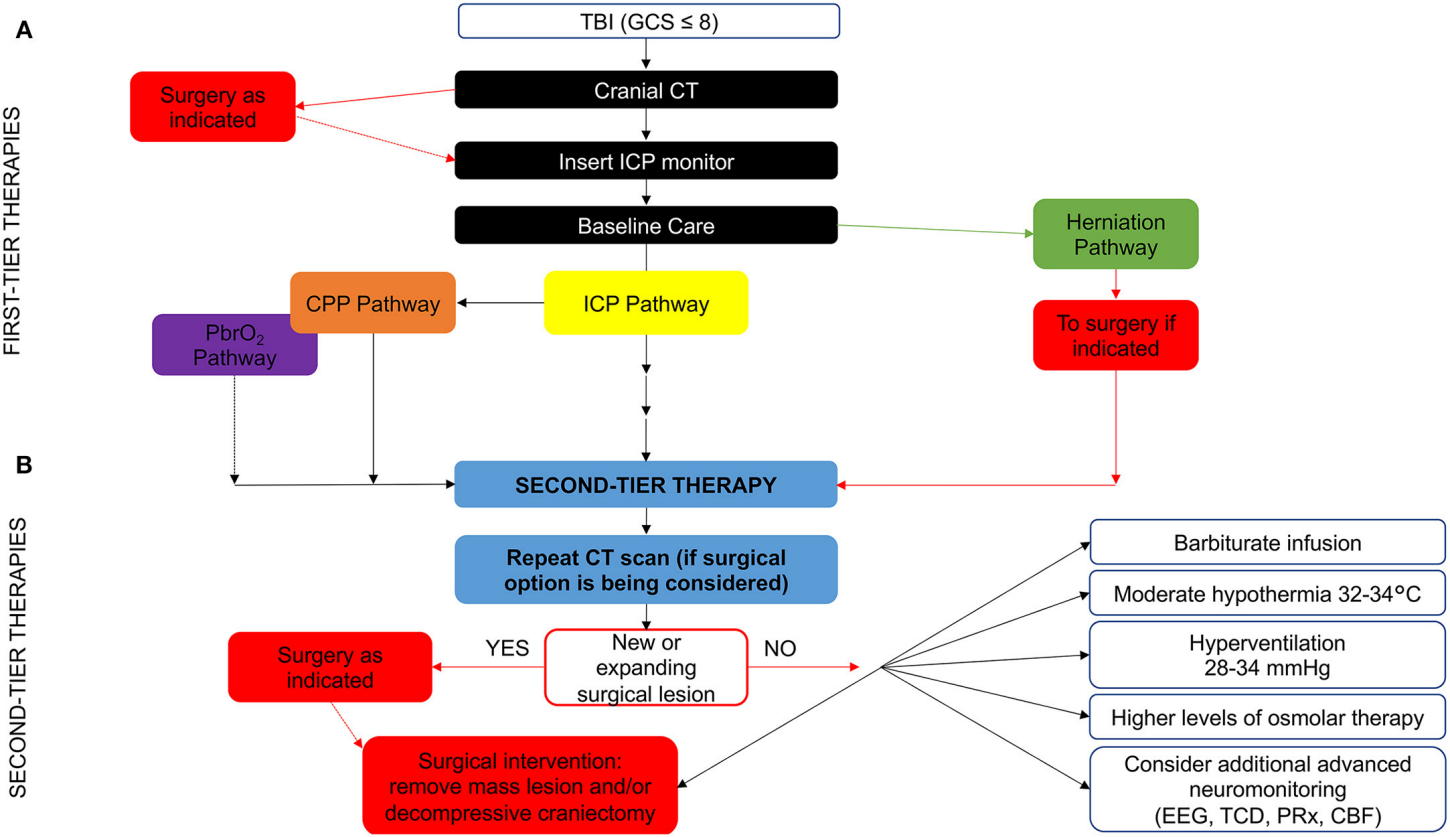
Evidence- and consensus-based algorithm of first- and second-tier therapies for pediatric TBI management. **(A)** The algorithm includes several components, such as baseline care (black), an ICP pathway (yellow), a herniation pathway (green), a CPP pathway (orange), and a PbrO_2_ pathway (purple). Solid lines identify the ICP and CPP pathways, reflecting their primary role, a dashed line identifies the PbrO_2_ pathway, given the fact that it represents a monitoring and management option, which is less commonly used and has less literature support. The caregiver should integrate all available information and implement the guidelines within the context of each patient's unique response to various therapies to establish the optimal treatment regimen. If baseline care is insufficient to control ICP, then progression down the ICP and CPP pathways is indicated (solid black line). First tier interventions progressing down the ICP pathway include optimized baseline care with controlled mechanical ventilation with normocarbia and adequate oxygenation, elevation of the head of the bed, nutrition, and appropriate analgesia and sedation, followed cerebrospinal fluid drainage and the use of hyperosmolar therapy as directed by ICP, CPP, or PbrO_2_. Please see the published TBI management algorithm (23) and Table 1 for details. The blue box indicates the need for second-tier therapy and represents the link to **(B)**, which represents the treatment options for refractory intracranial hypertension when tier 1 approaches are insufficient. These therapies may be applied singularly, serially or in combinations. In addition, as shown, the management of refractory intracranial hypertension in the second-tier phase may be aided by the use of advanced monitoring. Data are not available on the number of patients treated with each of the first and second tier therapies given that even Guidelines-based care is highly center dependent. CBF, cerebral blood flow; CPP, cerebral perfusion pressure; CT, computed tomography; EEG, electroencephalogram; GCS, Glasgow Coma Scale; ICP, intracranial pressure; PbrO_2_, brain tissue partial pressure of oxygen; PRx, pressure reactivity index; TBI, Traumatic brain injury; TCD, transcranial Doppler ultrasonography. Graphical elaboration of text and Figures 1, 2 in (23).

Another challenge to TBI guidelines development is the longstanding lack of consistency in patient care across centers, with <50% of clinical sites providing protocol-based care for children with severe TBI in PICUs (25). Thus, the BTF promoted the development of an algorithm as a practical attempt to help guide bedside care, as well as provided a framework for future research. The new algorithm focuses the many available treatment options given the low level of evidence for most of the guideline recommendations. The algorithm also aims to clarify important nuances of care (i.e., variations in “tempo” and timing during which therapies are implemented and the combining of different monitoring modalities, such as ICP and brain tissue oxygen tension (PbrO_2_) (Figure 1). While it is important to provide linear algorithm sequences for first-tier management of ICP, cerebral perfusion pressure (CPP) and PbrO_2_, the progression in first-tier may be not linear if multi-parametric monitoring is used. Nevertheless, refractory ICP can lead to progression through the entire first tier of therapy in a matter of hours or less (23).

Overall, the algorithm represents an approach to acute care that sets the stage for important medical, surgical and rehabilitation approaches in the subacute and delayed post-injury periods with the goal to optimize long-term outcomes in severe pTBI (23). It represents a guide to be tailored to each individual clinical case by placing more emphasis on the significance of the proposed thresholds that should be interpreted as “minimum therapeutic targets” rather than optimal targets.

While BTF guidelines recommend monitoring ICP to assess intracranial hypertension and direct therapy, in resource limited areas of the world there is no access to ICP monitor technology or when available (via ventriculostomy), it is rarely used. This is the consequence of multiple factors including limited availability of neurological surgeons, expense, complications, and labor intensity, among other reasons. This means that most pediatric patients with severe TBI are treated without use of ICP monitoring and may also not receive other forms of invasive monitoring. Early decompressive craniotomy without ICP monitoring in children with severe TBI and signs of raised ICP has been suggested in a case series of LMIC setting (26).

Unfortunately, there are no Guidelines and no literature on how to treat severe pediatric TBI without use of ICP monitors. A current trial (NCT02059941) is aiming at developing guidelines for the treatment of severe TBI in the absence of ICP monitoring and test them by working with a team of clinicians that practice in austere environments in LMICs and routinely make decisions based either on a treatment protocol, their clinical experience, or both. A recent Delphi survey involving a large multidisciplinary group of experienced neurosurgeons and intensivists from resource-limited countries identified a set of predictors derived from basic clinical and imaging findings that defined those patients whom they would treat for suspected intracranial hypertension in the absence of ICP monitoring (27). Furthermore, although a consensus-based guideline for severe TBI treatment without ICP monitoring has been developed by a 47-clinician task force, representing 15 Latin America countries, who routinely manage patients with severe TBI without monitors (28), no specific indications for pediatric patients were available. This reinforces the need to develop and implement both Guidelines and consensus-based clinical protocols and therapeutic options useful in LMICs in the absence of advanced monitoring tools.

Summary of gaps and actionable suggestions

- No level I recommendations are currently available for pTBI management
- A high variability in treatment algorithms, particularly for topics with limited evidence (es. ICP) exists among centers worldwide
- New comparative effectiveness studies may support further refinement of current recommendations (potentially useful also in LMICs)
- Tailoring treatment algorithms to each patient's needs as well as interpreting treatment thresholds as minimum therapeutic targets rather than optimal targets may optimize pTBI care (useful also in LMICs).

### Advanced Neuro-Monitoring

- ICP and related parameters

ICP and CPP monitoring are the current standards to guide therapy in severe TBI (22). However, clinical evidence supporting the link between ICP monitoring and improved outcomes remains poor and even less robust in children than in adults (22, 29–32). Several reasons may have contributed to this. First, ICP and CPP are inadequate as tools to inform clinicians efficiently about the complex brain pathophysiology underlying pTBI because of the multifactorial contributions to intracranial hypertension (33). Very dissimilar clinical conditions may result in very similar, yet unpredictable, variations in ICP that may not follow linear relationships as observed for the prolonged lag time existing between ICP variation and metabolic dysfunction, failing to produce the expected outcomes.

Second, studies in pTBI are also scant and suffer from small sample size, further inducing the use/extrapolation of adult thresholds. However, this fails to consider the subtle differences in anatomy and physiology between adults and children and even across pediatric age strata. Furthermore, in children, defining adequate ICP and CPP control is more complex than that in adults, because of different and less well-established normative thresholds for ICP and blood pressure (34, 35). Thus, we may need to tailor thresholds to age, baseline values, injury phenotype (diffuse or focal), and the therapeutic approach (i.e., the ICP threshold to administer hyperosmolar therapy likely differs from that used to perform a craniectomy) (36).

Third, the optimal approach to CPP-directed therapy appears to depend on the state of blood pressure autoregulation of cerebral blood flow (i.e., intact vs. impaired). Some have suggested the ability to delineate optimal CPP for patients with TBI, although not with universally validated methods (37). In this context, pressure reactivity index (PRx) can be used to evaluate cerebral autoregulation and by plotting average PRx across different CPP values, it is possible, in some patients, to estimate optimal CPP, namely the CPP at which the autoregulation is maximal (38, 39). However, although representing the best physical correlation between ICP and mean arterial blood pressure (MAP), “optimal CPP” may not represent the best perfusion pressure for a given patient with a specific pathophysiology, and issues of global vs. focal injury may further complicate the ability to define a truly optimal CPP. Nevertheless, to our knowledge, no established protocol exists for the management of brain swelling in severe pediatric TBI without an ICP monitor. And any such protocol or approach would almost certainly be based on prior clinical experience surrounding the result of such therapies on ICP and/or CPP (40).

Finally, many secondary injury pathways can operate independently of raised ICP, including neuronal death cascades (i.e., apoptosis, ferroptosis), facets of neuroinflammation, and synaptic damage, among others (41–43).

PbrO_2_ is used in a selected number of centers as a complementary tool to ICP monitoring and correlates with disease severity, mortality and metabolic alterations in TBI (44–46). However, debated treatment thresholds and two main technical limitations (catheter positioning and spatial resolution) hamper its use to direct optimal care in pTBI. First, catheter misplacement is very common and probe placement may sometimes require craniotomy; nevertheless, some neurosurgeons report that a craniotomy actually complicates probe placement. The tunneled probe has a higher propensity to shift position when the skin flap is returned. Second, because of its low spatial resolution, PbrO_2_ may reflect the oxygenation of a specific brain area failing to provide a comprehensive picture of the potential ischemia resulting from TBI (47, 48).

To date, the aforementioned monitoring tools, when considered individually, may vary and their changes are linked and dependent on a multifactorial variety of physiological and clinical parameters in children. Thus, an advanced neuro-monitoring approach that integrates ICP, CPP, PbrO_2_, and possibly other parameters, in a patient-tailored framework is needed to guide appropriate decision-making processes (49).

- Imaging and radiology

CT is the modality of choice for most initial assessments of severe TBI due to its ease of access, rapid acquisition and its sensitivity to detect skull fractures and acute hemorrhagic lesions that may require surgical intervention (50, 51). Management of the injured child requires special considerations and, among the issues unique to children, there is the need to limit radiation exposure as low as reasonably possible (52, 53). Clinicians should, thus, balance the risk of missing a potentially devastating intracranial injury vs. the risk of radiation exposure particularly in mild TBI cases that infrequently require CT (54). A repeat CT scan >24 h after the first assessment is not suggested to guide decisions on neurosurgical treatment, unless there is either evidence of neurologic deterioration or increasing refractory ICP (22).

The development of clinical prediction rules may optimize clinical management by reaching the optimal trade-off between patient risks and benefits. The Pediatric Emergency Care Applied Research Network (PECARN) is the only clinical prediction rule, including two different age-appropriate clinical assessments (<2 and ≥2 years), on the premise that children under 2 years have a different brain injury risk profile, have a different development-dependent ability to express signs and symptoms and are more sensitive to the effects of radiation from CT (50, 54, 55). Physicians should use the age-appropriate PECARN algorithms to help decision-making about head CT scans in children with a GCS ≥14 while favoring initial observation over CT for children at intermediate risk for clinically important TBI (54).

Although magnetic resonance imaging (MRI) does not expose children to ionizing radiation, conventional MRI requires the child to remain motionless for several minutes and usually requires anesthesia or sedation. MRI is typically reserved to detect lesions that may explain clinical symptoms, which remain unresolved despite initial CT, especially in the setting of diffuse axonal injury (DAI) (51, 56). Compared with CT, MRI provides prognostic insights and identifies significantly more intra-parenchymal lesions in pTBI, particularly in children AHT.

Promising alternative imaging modalities have recently emerged and bypass the long-term sedation and radiation risks. Fast sequence MRI (fsMRI) should be considered for pediatric patients with potential TBI to decrease radiation exposure while maintaining comparable diagnostic accuracy to CT (sensitivity of 93%). The ability to complete imaging in ~5 min, by using abbreviated sequences, suggests that fsMRI could be appropriate even in the ED, where patient throughput is a priority (57). MRI availability, staff expertise in facilitating non-sedated studies, and buy-in from neurosurgical colleagues and skilled radiologists to perform timely image reviews are all required to successfully implement fsMRI. Overall, fsMRI increased sensitivity for DAI but reduced sensitivity for non-depressed linear skull fractures (57). Further evidence is needed to establish MRI markers to prognosticate and guide therapy after severe pTBI.

Point-of-care ultrasonography (POCUS) or near infrared spectroscopy are increasingly used as adjunct diagnostic tools in the initial assessment of pTBI. US is fast, safe, portable, can be performed at the bedside, cost-effective and well-tolerated, even by children. POCUS can detect the type and depth of skull fractures, thus prioritizing patients for CT scan and earlier neurosurgical consultation. Nevertheless, the many limitations to the potential scope of the utility of these tools in clinical decision-making along with user-dependence of the findings stand as main drawbacks to their routine use (57, 58). A new approach, ultrasonography (US) assessment of the optic nerve sheath as a surrogate marker of brain edema, has emerged as an area of exploration that if validated, could have potential utility at the bedside, particularly in LMICs (59).

Along with CT and MRI, neurophysiological tools, such as electroencephalogram (EEG) allow the detection of the epileptogenic risk and most notably, can identify sub-clinical seizures or status epilepticus. The American Clinical Neurophysiology Society guidelines recommend routine EEG monitoring in severe pTBI, as the occurrence of electrographic seizures after severe TBI is higher in children than adults, occurring in up to 70% of cases (60). This is particularly true in infants and victims of AHT. Evidence supports the use of EEG throughout the management course, particularly when neuromuscular blockade is used or to detect electrical asymmetries. Anti-epileptic drugs are currently administered as seizure prophylaxis early after severe pTBI. Phenytoin and levetiracetam are the most commonly used medication (61). However, EEG monitoring, seizure prophylaxis, the management of status epilepticus or refractory status epilepticus, as well as the measurement of anti-convulsant drug levels vary greatly between centers as reported in ADAPT trial and PEGASUS (Pediatric Guideline Adherence and Outcomes) Study, likely reflecting the paucity of available data to guide clinical decisions (23, 61, 62).

Summary of gaps and actionable recommendations

- ICP and CPP are inadequate as tools to inform clinicians efficiently about the complex brain pathophysiology underlying pTBI because of the multifactorial contributions to intracranial hypertension
- Despite its use as a complementary tool to ICP, PbrO_2_ displays debated thresholds and two technical limitations (catheter positioning and spatial resolution) hamper its use in directing optimal care in pTBI
- Although conventional MRI does not expose children to ionizing radiation, CT remains the modality of choice for most initial assessments due to its ease of access, usually not requiring anesthesia or sedation.
- An advanced neuro-monitoring approach that integrates ICP, CPP, PbrO_2_, and possibly other parameters, in a patient-tailored framework is needed to guide appropriate decision-making processes
- The PECARN clinical prediction rules may optimize clinical management by reaching the most convenient trade-off between patient risks and benefits for mild pTBI (useful also in LMICs)
- fsMRI appears a valuable and feasible alternative to CT scan in the emergency department setting to reduce radiation risk related to CT scan.

### Decompressive Craniectomy and Cranioplasty: When, How, and Complications

Decompressive craniectomy (DC) is recommended in pTBI to treat neurological deterioration, herniation or refractory ICP at level III recommendations. The difference between adult (level IIa) (63) and pediatric recommendations may reflect the low quality and quantity of evidence on the use of DC in children (class III studies, case series with non-comparable designs) (22). Despite efforts to derive reliable conclusions, strong evidence is lacking on the role of DC in pTBI particularly for very young children, with current recommendations being partially adapted from adult studies.

The availability of higher quality data (i.e., the complete data for young patients) from the Randomized Evaluation of Surgery with Craniectomy for Uncontrollable Elevation of Intracranial Pressure (RESCUEicp) trial and the planned Decompressive Craniectomy for Severe TBI in Children with Refractory Intracranial Hypertension (RANDECPED) 2019 trial (NCT03766087) may inform stronger recommendations.

The RESCUEicp trial showed that, when used for refractory ICP, DC could be life-saving but resulted in higher rates of vegetative state and severe disability (64). However, by 12 months, *post-hoc* analysis revealed that the subset of younger patients in the DC group exhibited better functional outcome vs. the medical therapy group (65, 66). Despite its efficacy to control ICP and reduce mortality in severe TBI, DC is associated with significant early and late complications, including expansion of contusion volume, fungus cerebri, hemorrhagic infarction, seizures, infection, hygroma, and hydrocephalus (22, 65, 67).

The timing and technique of DC in pTBI have been a matter of lively debate. A primary DC is usually undertaken “as soon as possible” (done at the time of mass lesion evacuation), while secondary DC is driven by ICP. An ICP threshold of 20 mmHg is generally used (22); nevertheless, age-specific ranges are yet to be determined for children. As per DC technique, unilateral (focal lesion) and bifrontal DC (i.e., diffuse injury) represent the preferred surgical options, while bilateral circumferential and bilateral frontotemporal craniectomies are less frequently used (60, 61). No further specific recommendations for the technique of DC are available except an adequate decompression size including a wide opening (22, 65). A large fronto-temporo-parietal DC (not <12–15 cm diameter) was also recommended (63).

The optimal timing, the most appropriate surgical technique, and the specific benefits of DC for children are yet to be well-studied. Thus, the decision-making requires careful assessment by a multidisciplinary team (neurosurgeons, neuro-intensivists), and appropriate parental involvement to clearly outline the surgery pitfalls when discussing prognosis (67).

Finally, the observation that <10% of the RESCUEicp population was enrolled in LMICs, where TBI burden is substantial and the paucity of the neurosurgical workforce and absence of rehabilitation services impede adequate TBI care, raises the question on the feasibility, on a global scale, of recommendations on DC mostly derived from studies performed in HICs (68). In the aforementioned study of TBI in four LMICs in Africa, neurosurgical interventions were available in three out of the four centers and were performed in 16% of the cases (21).

The goals of cranioplasty following DC are to protect the brain and to improve neurological outcome with an optimal functional and aesthetic result. Usually, it has taken several months after DC to allow for recovery and to ensure that cerebral edema has subsided (69). Although these strategies have been extrapolated from those routinely practiced in adults, they may be not suitable or tailored for children. Furthermore, the patient age, normal skull growth and brain development could impact the available options. The optimal timing for cranioplasty for pTBI remains unclear. Early cranioplasty (from 30 days to 6 weeks after DC) reduces the risk of complications and improves the neurological outcome (70); however, it is not indicated in the presence of infection. Late cranioplasty (>6 weeks to 6 months) is associated with CSF disturbances (hygroma and hydrocephalus) and bone flap resorption (71, 72). In contrast to adults, autologous bone is still considered the “first choice” in children, given its biocompatibility and genetic match to the patient skull. Nevertheless, a significant incidence of complications, such as infection and bone resorption, resulting in high replacement rates (as high as 45.5% in infants) are frequently reported (71, 73–77). Furthermore, adequate, well-vascularized soft tissue is an important factor to achieving successful reconstruction. Indeed, the small thickness of the bone flaps, the poorly represented diploe (78), and the size of the original skull (73) may enhance the resorption rate (73), as can the use of extra-cranial donor bone (i.e., ribs, iliac crest) rather than skull bone (77, 78).

Custom-made implants have recently gained interest, resulting from the introduction of different alloplastic materials: metals (titanium), acrylics (poly-methyl-methacrylate), plastics (poly-etheretherketone), and ceramics (hydroxyapatite). Cranioplasty-related costs differ among various materials and this should be considered in both LMICs and HICs with state-funded healthcare systems. If the skull defect is large or bone flap is inadequate, custom-made implants can be considered for patients older than 6 months and with less risk of a second trauma (78–80). Notwithstanding their effectiveness in reducing complications (14.2 vs. 36.2%), it is a matter of debate if implanting an inert prosthesis may be burdened by drawbacks in the long-term follow-up (70–72). Current research is also focusing on materials with osteoconductive and osteoinductive properties. If the implant is well-osteo-integrated it may have a lower rate of displacement, skin damage and flap removal (78–80). Further evidence is needed to identify the optimal allograft materials for cranioplasty, the age-related factors affecting cranioplasty outcomes, as well as to clarify the secondary disorders of CSF dynamics.

Summary of gaps and actionable recommendations

- No strong evidence is yet available regarding the role of DC in pTBI particularly for very young children, with current recommendations being partially adapted from adult studies
- The optimal timing, the most appropriate surgical technique, and the actual benefits of DC and cranioplasty for children are yet to be well-studied
- The optimal timing for cranioplasty for pTBI remains unclear
- A careful assessment by a multidisciplinary team (neurosurgeons, neuro-intensivists) should be promoted in clinical practice along with appropriate parents' involvement to clearly outline DC pitfalls when discussing prognosis
- Pending further research on materials with osteoconductive and osteoinductive properties, studies are needed to identify the optimal allograft materials for cranioplasty and the age-related factors affecting cranioplasty outcomes.

## Short- and Long-Term Outcomes

Outcomes after pTBI span the spectrum from full recovery to behavioral and psychosocial problems, deficits in communication, deficits in daily living skills and general adaptive function, and severe disability or death. The mortality rate in severe TBI ranges from 16 to 22% (81, 82); however, children with severe TBI presenting with a GCS score of 3 or 4 have a higher likelihood of death (>50%) and morbidity (83).

Plasticity of the immature brain has been proposed to facilitate adaptations to initial insults, leading to improved overall outcomes or even survival. Alternatively, some argue that an injury during a critical early developmental stage may disrupt key developmental processes (84). As these developmental processes unfold over time, many additional variables must be assessed to identify how age affects outcome (8, 85).

The chronic effects of TBI may be greatly impacted by developmental processes involved in brain maturation, thus revealing some symptoms only when the child has reached certain level of developmental maturation. As a result, in many children with mild TBI, behavioral and neuropsychological changes are often underestimated. Furthermore, given the underestimated link between intracranial injuries and the development of chronic neurodegenerative diseases (i.e., chronic traumatic encephalopathy, memory loss), the long-term burden of TBI in children is likely underestimated (86). Only few studies have investigated the impact of pTBI on long-term outcomes (10 years or more) post-injury. While injury-related factors cannot be changed, fortunately, trajectories of chronic outcomes can be influenced positively by providing young patients with cognitive and behavioral support while training parents with supportive intervention strategies (83, 87).

**Figure 2:**
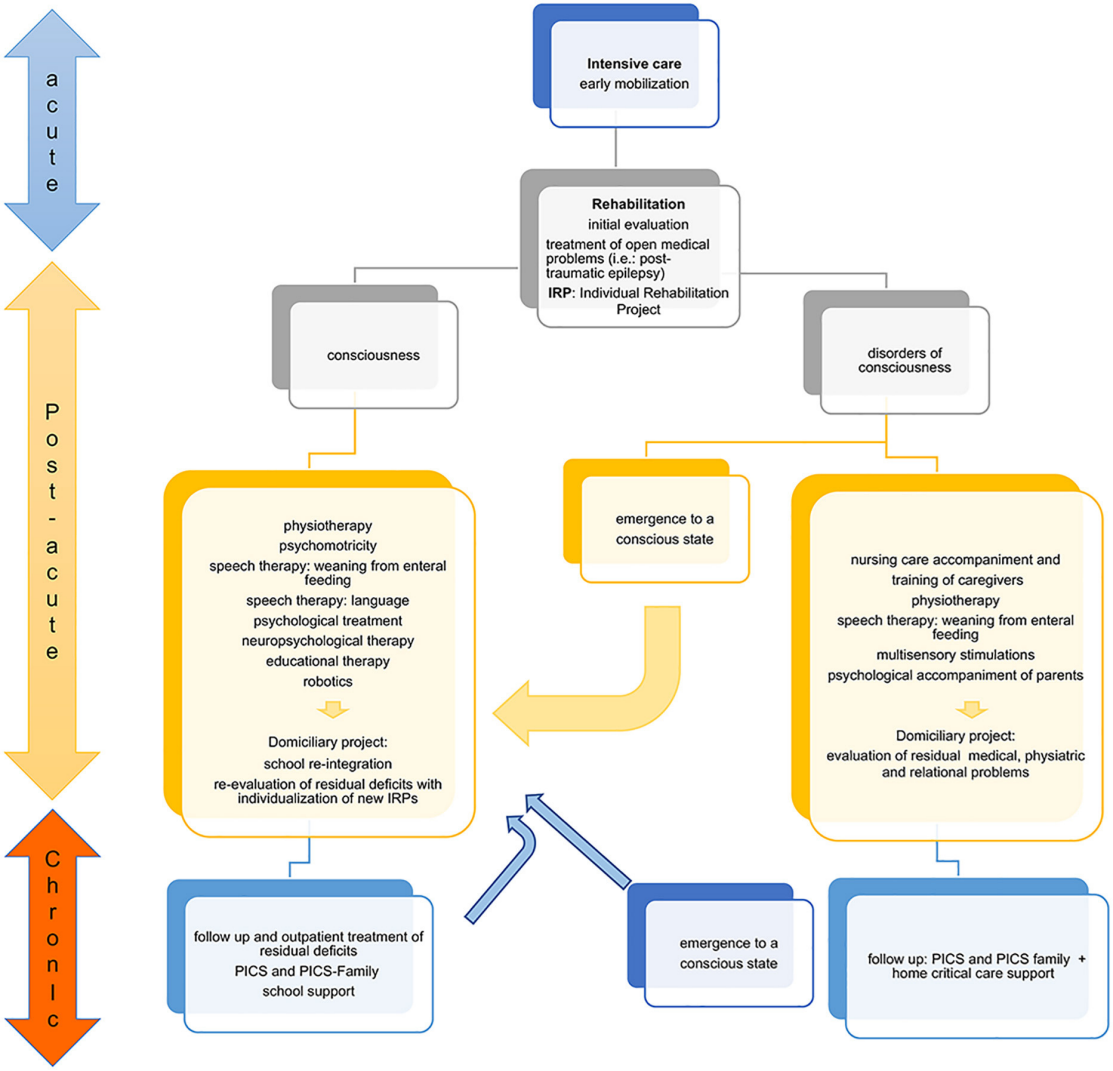
Flowchart of clinical recovery and main outcomes during the rehabilitation process. The patient who comes out of intensive care must be appropriately assessed to set up the Individual Rehabilitation Project (IRP). The state of consciousness conditions two different rehabilitation paths. In the conscious patient, the goal is to recover the impaired functions. In the patient with a disorder of consciousness the main goal is to stimulate the state of consciousness. The IRP requires continuous re-evaluation and updating because the patient can emerge from the state of consciousness both in the post-acute phase, and more rarely, in the chronic phase. In this case, an intensive rehabilitation program is useful. The child with functional recovery must be re-evaluated during the development and modification of the functional deficit, as new rehabilitation objectives may emerge that require a new intensive path. Post-intensive care Syndrome (PICS). The proposed approach, arising from the experience of Bosisio Parini Institute, could be generalizable to other experience.

Figure 2 summarizes the evolution of clinical/neurological impairments, medical/rehabilitation needs and educational status during rehabilitation programs. The efficacy of a rehabilitation program could be valuable in the short and medium-term (from 1–3 months to 1–2 years after injury). While improvement in cognitive domains often occurs during the recovery phase post-injury, residual deficits often persist in the chronic phase (2 years post-injury or later). Attention, processing speed, memory, and executive functions represent the cognitive domains mainly affected in the chronic stage of recovery (88).

Given the complex interaction between individual and context factors and outcome, the panel of international expert of the congress focused on six areas of interest that could significantly impact the outcome even in LIMC setting: prognostic models, CER, patient and family-centered care, rehabilitation, rehabilitation programs, organizational models of care.

### Prognosticating in pTBI

Accurate prediction of outcome after moderate-to-severe pTBI is essential to guide family expectations and improve the effectiveness of rehabilitation. However, predicting prognosis after pTBI is challenging due to heterogeneity of patient- or injury-related factors, outcome measures and research methodology. TBI affects multiple outcome domains, thus focusing mainly on mortality and dichotomous extended Glasgow Outcome Scale (GOSE) score (named GOSE-Pediatric [GOS-E-Peds] in children) fails to fully describe the range of pTBI outcomes. Given the relevant incidence of neurological disabilities after pTBI, other outcomes measures, including cognitive and neuropsychological tests, must be prioritized to better define the burden of illness (87, 89). The failure to select measures that would be appropriate for children and infants is another notable limitation. The Functional independence measure for children (Wee-FIM), composed of 3 subscales rating self-care, mobility and cognitive abilities, could be proposed as a comprehensive measure of children and youth functional status in the short- and long-term (88, 90). Nevertheless, the heterogeneity in study methods and neuropsychological measures, as well as differences between center standards of care limit collection and comparison of outcome data post-pTBI (25, 89).

Predictive models, such as Pediatric Index of Mortality (PIM) and Pediatric Logistic Organ Dysfunction (PELOD), are commonly used to discriminate mortality risk in PICU, but their performance in terms of calibration and discrimination has never been assessed in pTBI (91).

Other tools to aid in outcome prediction in pTBI are emerging, including the use of serum biomarkers of brain injury. Early studies suggested potential capability of serum biomarkers such as neuron specific enolase (NSE), glial fibrillary acidic protein (GFAP), S100B, and Ubiquitin C-terminal hydrolase-L1 (UCH-L1), including studies in pTBI showing their ability to differentiate accidental from AHT and brain injury from asphyxia (92). These biomarkers have potential also as diagnostic tools particularly in mild TBI and to monitor response to therapy. Recently, GFAP and UCH-L1 were FDA approved in the United States for use as diagnostic adjuncts in mild TBI (93). Additional discussion of this emerging area is beyond the scope of this review (94).

### Prognosticating in pTBI Using Comparative Effectiveness Research to Identify Best Practices and Improve the Quality of Care in pTBI

CER has special potential in the field of TBI. While randomized controlled trials (RCTs) have currently failed because of marked heterogeneity in patients, injury-specifics, and background care, CER takes advantage of heterogeneity in care to generate evidence in TBI, by analyzing current guideline adherence, treatment variation and therapies associated with the best outcomes (25). The concept of a CER study using a large-scale observational design, as implemented in the InTBIR (International Initiative for Traumatic Brain Injury Research), has resulted in a rising number of linked collaborative projects.

The CREACTIVE study (Collaborative ResEarch on ACute TBI in intensiVe care medicine in Europe) is a large network of ICUs across seven European countries and has enrolled >7,000 TBI patients of which 795 are children. It aims to better describe the epidemiology of moderate–severe TBI, build a prognostic model based on short- and long-term outcome measures, identify the most effective clinical interventions to optimally treat TBI patients, and recognize the determinants of optimal vs. suboptimal performance (https://cordis.europa.eu/project/id/602714/it; http://creactive.marionegri.it/).

The ADAPT trial is a CER study of children with severe TBI, enrolling 1,000 children from over 51 centers and has as an inclusion criterion the placement of an ICP monitor. It is aimed to assess the effectiveness of treatments on relevant outcomes across severe pTBI to ultimately improve healthcare (8) and to address questions that have eluded examination via RCTs. It is also an important demonstration that part of the value of a guideline is rooted in both the evidence-based recommendations and in highlighting what cannot be said due to lack of evidence.

### Patient and Family-Centered Care in pTBI

There is a mounting need to design models of care aimed at implementing family-centered care (FCC) to support better outcomes in critically ill children and their families (94). Parents experience a switch in their role from being the person responsible for the safety and care of their child to being completely reliant on the PICU medical team. This is coupled with countless other stressors, including witnessing the pain, fear and often shocking physical changes in their child and facing many difficult decisions (94). A key element of care delivery models in pediatric settings is recognition of the importance of parent/caregiver participation. Prior studies have linked patient and family-centered care (PFCC) to improve patient clinical outcomes, family and staff satisfaction and reported positive changes, such as reduced parental-reported anxiety and improved communication between parents/caregivers and health professionals (94).

PFCC encompasses four core concepts: respect and dignity, information sharing, participation in care and decision-making and collaboration between patients, family and healthcare system (95, 96). An integrated and multidisciplinary approach has been applied to make the family experience as much positive as possible. Effective communication and participation in decision-making empower parents with knowledge and skills to be an active part of the care provision, improve policy, and reduce distress, distrust, risk of judicial litigation and healthcare resources. Finally, family members should be actively included as collaborators in rehabilitation and reintegration to home, school, work and community life.

### Rehabilitation Programs and New Concept

Advances in the acute management of TBI reduce mortality while increasing rehabilitation needs in the survivors. However, summarizing the rehabilitation literature for outcomes after pTBI is challenging due to various methodological limitations, disparities in access to rehabilitation, limited awareness of deficits and the lack of services in the local setting (97), even if an intensive rehabilitation is required (Figure 2).

The optimal time to begin rehabilitation and the early mobilization of the critically ill pTBI patient is an important issue (98, 99). The feasibility, efficacy, safety and financial benefits of a pediatric Early Mobility (EM) Program in the PICU have been reported. The time from PICU admission to mobilization can be decreased from 20 to 14 h with 84% of patients being mobilized within the “allotted” time frame (i.e., 18 h for non-mechanically ventilated and 48 h for mechanically ventilated patients). TBI can lead to a wide range of functional impairments. Each TBI child would need an assessment to define rehabilitation priorities. To this respect, individual's program must be designed to address TBI-related rehabilitation needs. When assessing the impact of rehabilitation, one should follow the neurological evolution of the patient and the long-term behavioral recovery. A motor recovery may not be accompanied by a comparable recovery in terms of preserved HRQoL, satisfactory social integration and return to daily living. These observations highlight the clinical relevance of prolonging neuropsychological and motor rehabilitation for years after TBI and monitoring long-term results, especially in those children at higher risk of neuropsychological disabilities (100, 101).

TBI can lead to a broad spectrum of functional impairments. Anticipating the long-term outcome trajectory aids the experienced clinician in establishing priorities and optimizing the approach to tailoring the rehabilitation needs to the patient. Different methodologies may be required, and when appropriately applied, provide synergy. For example, this occurs for the functional recovery of the hemiplegic hand where traditional, innovative and robotic methods can be used (102). After intensive rehabilitation, the child must be followed in order to be helped in the developmental stages. This can happen with outpatient rehabilitation or with more innovative tele-rehabilitation courses.

Family advisors can be included in the multidisciplinary team to both assess family knowledge on EM/ rehabilitation benefits and incorporate family perspective and feedback, thus enhancing PFCC (103). Research on early (ICU-based) rehabilitation for pediatric neurocritical care patients appears to be feasible; effectiveness trials are needed to determine impact on outcomes (104).

### Organizational Models to Improve Outcomes

Infrastructure, processes of care and outcome measurements are the cornerstone of quality care for pediatric trauma, with the organization of critical care services being another relevant determinant of quality. At the core of the organization of trauma care is the level I trauma center, which is mostly located at referral centers in major urban environments. A concentration of resources in level I and II trauma centers along with a marked regionalization of care provision may reduce pTBI mortality. Nevertheless, substantial variations in both provision of care and short- and long-term outcome exist between centers and tackling these differences holds great promise to improve TBI management across the continuum of care. Quality indicators by phase of care have been identified in pre-hospital care, in-hospital and post-hospital care as well as family/care-taker burden (105) and based on these, we propose the desired conditions and the most common areas for improvement to ensure that injured children receive appropriate emergency care (Table 2). This information may inform educational interventions, as well as promote research program development. A highly collaborative and mutually respectful team is required throughout the continuum of care, to provide cohesive and efficient treatment and maximize all facets of outcome. Establishing such a collaborative approach also facilitates research opportunities and productivity.

**Table 2:** Organizational models for pediatric trauma centers: desirable conditions and most common areas of improvement.

**Desirable conditions**	**Areas of improvement**
**Pre-hospital care**	
Availability of HEMS services Development of trauma assistance integrated system Centralization of the provision of care by directing patients to level I trauma centers Dedicated pediatric transport system for all inter-hospital transports with ability to support far outlying hospitals without dedicated pediatric surgery and anesthesiology staff Activation of the trauma team Permanent prevention education dedicated to parents and caregivers	Reduce admission of moderate-to-severe pediatric TBI patients to adult trauma units Increase availability of ICU ambulances Improve the ratio of 1 neurosurgeon:2.5 million patients in LMICs Reduce the incorrect application of centralization protocols Optimize medical pediatric training and airway management
**Emergency room**	
24/7 availability of senior physician expert in pediatric trauma care Optimized coordination with the national emergency service Presence of psychological support in red room Permanent simulation program Record time to CT (<20 min) and to ICU (<1 h)	Increase both the time for training and medical education, and its quality Ensure the presence of pediatric trauma specialist Include dedicated on-call residents Standardized use of rapid infuser and intracranial monitors Increase ICU bed capacity
**Monitoring**	
ICP/CPP driven protocols ICP bolt/EVD can be inserted at the bedside Monitoring of intracranial compliance CSF pharmaco-metabolomics Early MRI application in AHT HB transcutaneous monitoring Formal training for board certification in pediatric neurocritical care	Increase availability of PbrO_2_ or micro-dialysis catheters Build an MRI suite in or near to the PICU and or enhance transport safety for current MRI locations Expand use of NIRS outside of the cardiac ICU Begin to implement and study the routine assessment of PRx Expand training programs in pediatric neurocritical care
**Family engagement**	
Adoption of PFCC Parents' presence at the bedside from PICU admission Daily meeting with the multidisciplinary team Active parental participation Comfortable environment Presence of child protective service investigative team	Increase resources for the caring parent Expand the space at the bedside Develop a programmatic assessment of the quality of life and post-traumatic stress disorder in parents Include psychologist involvement in the PICU Develop outreach for social services and support to families who lost their loved one at adult facilities before transport to the pediatric center
**Rehabilitation**	
Adoption of the early rehabilitation protocol Physical and occupational therapy during PICU admission Rehabilitation consultation before discharge Speech and language therapy consult Effective follow-up when discharge at home	Include a dedicated pediatric rehabilitation specialist in hospital Develop on-site rehabilitation and structures that bridge in-hospital rehabilitation to outpatient rehabilitation Increase time available for rehabilitation in-house bed (weeks to months) Serially evaluate for unmet needs Coordinate with primary care physician and school

*AHT, abusive head trauma; CPP, cerebral perfusion pressure; CSF, cerebrospinal fluid; CT, computed tomography; EVD, external ventricular drain; HB, hemoglobin; HEMS, helicopter emergency medical services; ICP, intracranial pressure; ICU, intensive care unit; LMICs, low–middle income countries; MRI, magnetic resonance imaging; MRS, magnetic resonance spectroscopy; NIRS, near infrared spectroscopy; PbrO_2_, brain tissue oxygen tension; PFCC, patient family-centered care; PICU, pediatric intensive care unit; PRx, pressure reactivity index; TBI, traumatic brain injury*.

Summary of gaps and actionable recommendations

- Given the underestimated link between intracranial injuries and the development of chronic neurodegenerative diseases (i.e., chronic traumatic encephalopathy, memory loss), the long-term burden of TBI in children is likely underestimated
- Prognosticating after pTBI is challenging due to heterogeneity in patient- or injury-related factors, outcome measures and research methodology
- CER may inform prognostic model development in pTBI, current guidelines adherence, treatment variation and therapies associated with the best outcomes (useful also in LMICs)
- Identification of reliable and predictive scores to monitor both the historical outcome (i.e., mortality) and functional (assessed by GOSE-P score) and cognitive domains is key to assess pTBI long-term outcomes
- Trajectories of chronic outcomes can be influenced positively by providing young patients with cognitive and behavioral support while training parents with supportive intervention strategies (useful also in LMICs)
- The assessment of rehabilitation outcomes post-TBI is challenging on a global scale due to various methodological limitations, disparities in access to rehabilitation, limited awareness of deficits, and the lack of services in the local setting
- Pediatric patients with TBI should be assessed to define rehabilitation priorities. In addition, each individual's program should be designed to address TBI-related rehabilitation needs. When assessing the impact of rehabilitation, one should follow the neurological evolution of the patient and the long-term behavioral recovery (useful also in LMICs)
- The development of an intensive care unit that implements a family-centered care is emerging as an area of improvement in the analysis of organizational models (applicable also in limited resource settings).

## Education and Research Priorities to Optimize PTBI Management

The impressive rising burden of pTBI, with at least 20% of PICU admissions requiring the involvement of a neuro-critical care (NCC) service (106), demands for a greater number of pediatric neurosurgeons and intensivists than are currently available (neurosurgeon/patient ratio: 1:80,000 in HICs and 1:10,000,000 in LMICs) (107). There is a need to expand the current neurosurgical workforce by enrolling and effectively training the young generation of physicians and allied personnel to support this field from the neurosurgical, NCC and rehabilitative perspective, along with other key participants such as neuroradiology, electrophysiology, child neurology, nursing, respiratory therapy, and others (108, 109). Furthermore, care gaps and inconsistencies raise concern about inadequacies in TBI training for physicians and the need to improve education is consistently raised world-wide (Table 2).

Improved knowledge of the unique features of pTBI, such as type of injury, associated second insults (i.e., hypoxemia, hypotension, coagulopathy), age-related insults (i.e., AHT) high prevalence of subclinical and clinical seizure activity, type of skull fractures, and other factors, should comprise the core of future educational efforts (110). For example, children display a reduced cardiac reserve which may impact the approach to stabilization in the ED or during surgery.

Although progress has been made in understanding different aspects of the pathophysiology of pTBI, this has produced only incremental advances, and the number of RCTs of new therapies has been limited (111). Current neurosurgical therapeutic options are limited to relieving pressure (by evacuating hematomas, removing bone or draining CSF) and supporting the patient with NCC. The failure to translate therapies to clinical success in TBI may have stemmed from the marked heterogeneity of TBI pathology and patient characteristics, the adoption of the “one-size-fits-all” approach (that does not acknowledge all the features underlying the TBI natural history) and the lack of stratification of treatment based on endophenotype or CT scan. This has been true for both pediatric and adult TBI, and across the spectrum of injury severity. Finally, current clinical evidence from adult TBI studies provides limited additional guidance.

New approaches are urgently needed to successfully translate therapies and novel diagnostics (such as serum biomarkers) from the pre-clinical arena to therapeutic successes in clinical trials in the field of pTBI. Precision medicine-driven approaches along with novel TBI classification beyond GCS and anatomical phenotyping may all stand as promising avenues for future studies. The experience of the ADAPT trial and its upcoming findings point to CER and adaptive trial design as bona-fide research strategies to guide optimal therapy selection within the complex scenario of pTBI.

Summary of gaps and actionable recommendations

- Limited neurosurgical workforce and inconsistencies and gaps in TBI training exist at the global level (useful also in LMICs)
- The progress that has been made in understanding the pathophysiology of pTBI has not translated into clinical practice
- Precision medicine-driven approaches along with novel TBI classification beyond GCS and anatomical phenotyping may all represent promising avenues for future studies (useful also in LMCIs)
- Common methods and descriptors for collaborative research are urgently needed to address the paucity of pediatric trials and limited extrapolation of adult data to pediatrics (useful also in LMICs).

## Pediatric Trauma Care During Covid-19 Outbreak

The current worldwide COVID-19 pandemic threatens to affect the ability to care for critically injured pediatric patients. As of today, neonates and children seem to be relatively spared of severe symptoms of COVID-19, even though the reasons for such a phenomenon still remain unclear (112, 113). Nevertheless, in many countries we have witnessed a genuine humanitarian crisis (114). In such a dramatic situation, children and their families—especially the most vulnerable and fragile—quickly become the “hidden victims” of this crisis because limiting their care stands as a huge COVID-19 collateral effect (115). Thus, it is extremely important (116), to prioritize all the primary healthcare interventions. A major goal is to preserve pediatric trauma care without increasing the virus spreading. To this end, trauma and emergency general surgeons, as well as highly-specialized physicians should all be protected by appropriate personal protective equipment (PPE) because their contagion or death may result in the inability to address trauma emergencies during and after the outbreak (117). Patients who are not tested or test negative for acute infection should be assumed potentially infected with SARS-CoV-2 and thus potential vectors (118). Until the value of the serology test is established, one should consider any body fluid as virtually infectious and further adopt universal operating room respiratory precaution (118). During surgery and invasive procedures, such as intubation, it would be advisable to minimize the presence of staff in the room, strictly apply good hand hygiene and using full PPE (118). According to the principle of caution, patient flow and PFCC should be appropriately revised. Last, but not least, interventions aimed at providing health professionals and families with psychological support should all be prioritized (119). During the pandemic lock-down period, tele-rehabilitation has become the only means of access for many patients adhering to rehabilitation programs (120) and many rehabilitation centers were equipped to best address the challenges of families with disabled children (121), despite the fact that the evidence in the pediatric field is limited (122) Overall, there are several clinical experiences and methods, ranging from video calls to treatments. These take advantage of programs developed to be executed via teleconference and implement tools specifically designed for telerehabilitation (123). This health emergency must push us to evaluate the true effectiveness of these tools. Telemedicine capabilities are likely to be embedded within normal operations, scalable, interoperable and built on a strong, reliable infrastructure, so that they may serve as effective approaches even after the acute COVID-19 crisis resolution (124).

## Discussion

Named the “silent epidemic,” TBI contributes to worldwide neurological disability more than any other traumatic event and its burden appears disproportionate in LMICs compared with HICs, thus highlighting the need of both developing context-specific clinical practice guidelines and building global research collaborations to bridge the existing gaps in knowledge on all aspects of the provision of care. Despite the magnitude of its burden, high-quality data are limited to inform policies for prevention and appropriate allocation of healthcare resources. The large number of children with mild TBI suggests that preventative strategies to reduce incidence of mild injuries could have a major clinical impact while being highly cost-saving. In contrast, for severe pTBI, allocating more resources to improve access to care and support clinical trials both to define the best current therapy, and develop new diagnostics and therapies would be optimal. In this context, substantial gains could also be made from the provision of adequate pre-hospital care, appropriate referral and continuity along the care chain.

Appropriate triage impacts both short- and long-term outcomes after pTBI with survival improved with care delivered at pediatric trauma centers, vs. adult or adult trauma centers with pediatric qualifications, despite no apparent significant differences in ISS (125). Accordingly, children should be triaged preferentially to pediatric-capable trauma centers.

Critical gaps in knowledge of pathophysiological differences between children and adults concerning TBI outcomes, the paucity of pediatric trials and the uncertain extrapolation of adult data to pediatrics demand collaborative research. Better, simple, easy to distribute and analyzed, practical and universal for as many countries and communities—are of great need in order to have the ability to benchmark and compare different medical treatments and rehabilitation approaches and their effect on outcome. As prognostic models for pTBI remain in their infancy, greater emphasis should be placed on identifying reliable and predictive scores to monitor both the historical outcome, that is, mortality and functional (assessed by GOS-E-Peds score) and cognitive outcome.

Finally, to improve pTBI care worldwide, it is crucial that solutions in LMICs should be tailored to local needs and resource availability, rather than replicating strategies in HICs. Recent studies have begun to provide clues on research goals and targets in this regard (21). Furthermore, consensus-based best practices and research goals should be achieved by including LMICs neurosurgeons, pediatric intensivists, pediatric emergency medicine physicians, rehabilitation specialists, and all participants of the continuum of care.

## Collaborative Pediatric TBI Working Group

Guido Bertolini^1^, Ezio Bonanomi^2^, Silvia Bressan^3^, Osvaldo Chiara^4^, Giuseppe Citerio^5^, Anthony Figaji^6^, Ericka L Fink^7^, Alberto Gabrieli^8^, Simonetta Gerevini^2^, Maurizio Iacoangeli^9^, Isaac Lazar^10^, Luca Ferdinando Lorini^2^, Christian Matula^11^, Isabella Pellicioli^2^, Gianluca Piatelli^12^, Franco Servadei^13^, Mirco Sicignano^2^, Dennis W Simon^7^, Sandra Strazzer^14^, Giuliana Vitali^2^

^1^Istituto di Ricerche Farmacologiche Mario Negri IRCCS, Ranica, Italy; ^2^Papa Giovanni XXIII Hospital, Bergamo, Italy; ^3^University of Padova, Italy; ^4^ Universita' degli Studi di Milano - Chirurgia Generale Trauma Team ASST Niguarda, Milan, Italy; ^5^University of Milan- Bicocca, Italy, San Gerardo Hospital, Monza, Italy; ^6^University of Cape Town, Republic of South Africa; ^7^Safar Center for Resuscitation Research, John G Rangos Research Center, UPMC Children's Hospital of Pittsburgh, University of Pittsburgh School of Medicine, Pittsburgh, PA, USA; ^8^APSS Trento Hospital, Italy; ^9^Umberto I General Hospital, Marche Polytechnic University, Ancona, Italy; ^10^Soroka University Medical Center and the Faculty of Health Sciences, Ben Gurion University of the Negev Be'er Sheva, Israel; ^11^Medical University of Vienna, Austria; ^12^IRCCS Istituto Giannina Gaslini Children's Hospital, Genoa, Italy; ^13^Humanitas University, Rozzano, Italy; ^14^Scientific Institute, IRCCS E Medea, Acquired Brain Injury Unit, Bosisio Parini, Lecco, Italy.

## Author Contributions

MN and FF conceptualized, designed the study, and drafted the initial manuscript. MN, FF, FB, and RZ performed the literature search, reviewed the studies, and drafted the manuscript. TB, EF, DS, and PK critically reviewed the manuscript. All authors, including the Collaborative Pediatric TBI Working Group, approved the submitted version.

## Conflict of Interest

The authors declare that the research was conducted in the absence of any commercial or financial relationships that could be construed as a potential conflict of interest.

## References

[1] Maas AIR Menon DK Adelson PD Andelic N Nell MJ Belli A Traumatic brain injury: integrated approaches to improve prevention, clinical care, and research. Lancet Neurol. (2017) 16:987–1048. 10.1016/S1474-4422(17)30371-X 29122524

[2] GBD 2016 Traumatic brain injury and spinal cord injury collaborators Global, regional, and national burden of traumatic brain injury and spinal cord injury, 1990–2016: a systematic analysis for the global burden of disease study 2016. Lancet Neurol. (2019) 18:56–87. 10.1016/S1474-4422(18)30415-0 30497965 PMC6291456

[3] Tropeano MP Spaggiari R Ileyassoff H Park KB Kolias AG Hutchinson PJ. A comparison of publication to TBI burden ratio of low- and middle-income countries versus high-income countries: how can we improve worldwide care of TBI? Neurosurg Focus. (2019) 47:E5. 10.3171/2019.8.FOCUS19507 31675715

[4] Hofman K Primack A Keusch G Hrynkow S. Addressing the growing burden of trauma and injury in low- and middle-income countries. Am J Public Health. (2005) 95:13–17. 10.2105/AJPH.2004.039354 15623852 PMC1449844

[5] Figaji A. Anatomical and physiological differences between children and adults relevant to traumatic brain injury and the implications for clinical assessment and care. Front Neurol. (2017) 8:685. 10.3389/fneur.2017.00685 29312119 PMC5735372

[6] Haarbauer-Krupa J Ciccia A Dodd J Ettel D Kurowski B Lumba-Brown A. Service delivery in the healthcare and educational systems for children following traumatic brain injury: gaps in care. J Head Trauma Rehabil. (2017) 32:367–77. 10.1097/HTR.0000000000000287 28060211 PMC6027591

[7] Gross BW Edavettal MM Cook AD Rinehart CD Lynch CA Bradburn EH. Big children or little adults? A statewide analysis of adolescent isolated severe traumatic brain injury outcomes at pediatric versus adult trauma centres. J Trauma Acute Care Surg. (2017) 82:368–73. 10.1097/TA.0000000000001291 27805998

[8] The ADAPT Trial Available online at: www.ADAPTtrial.org.

[9] Dewan MC Mummareddy N Wellons JC Bonfield CM. The epidemiology of global paediatric traumatic brain injury: a qualitative review. World Neurosurgery. (2016) 91:497–509. 10.1016/j.wneu.2016.03.045 27018009

[10] Joyce T Huecker MR. Pediatric Abusive Head Trauma (Shaken Baby Syndrome). StatPearls Treasure Island (FL): StatPearls Publishing. (2020) Available online at: https://www.ncbi.nlm.nih.gov/books/NBK499836/ (accessed Jun 26, 2020).

[11] Meeuws S Yue JK Huijben JA Nair N Lingsma HF Bell MJ. Common data elements: critical assessment of harmonization between current multicenter traumatic brain injury studies. J Neurotrauma. (2020) 37:1283–90. 10.1089/neu.2019.6867 32000562 PMC7249452

[12] Quayle KS Powell EC Mahajan P Hoyle JD Nadel FM Badawy MK. Epidemiology of blunt head trauma in children in US emergency departments. N Engl J Med. (2014) 371:1945–7. 10.1056/NEJMc1407902 25390756

[13] Bekelis K Missios S Mackenzie TA. Pre-hospital helicopter transport and survival of patients with traumatic brain injury. Ann Surg. (2015) 261:579–85. 10.1097/SLA.0000000000000672 24743624 PMC4446980

[14] Elswick CM Wyrick D Gurien LA Rettiganti M Gowen M Pownall A Resource utilization and indications for helicopter transport of head-injured children. J Pediatr Surg. (2018) 53:1795–99. 10.1016/j.jpedsurg.2018.04.032 29792280

[15] Rose MK Cummings GR Rodning CB. Is helicopter evacuation effective in rural trauma transport? Am Surgeon. (2012) 78:794–7. 10.1177/000313481207800720 22748540

[16] Maissan IM Verbaan LA van den Berg M Houmes RJ Stolker RJ den Hartog D. Helicopter transportation increases intracranial pressure: a proof-of-principle study. Air Med J. (2018) 37:249–52. 10.1016/j.amj.2018.02.010 29935704

[17] Englum BR Rialon KL Kim J Shapiro ML Scarborough JE Rice HE. Current use and outcomes of helicopter transport in pediatric trauma: a review of 18,291 transports. J Ped Surg. (2017) 52:140–4. 10.1016/j.jpedsurg.2016.10.030 27852453

[18] Global status report on road safety 2018: summary Geneva: World Health Organization (2018). (WHO/NMH/NVI/18.20). Licence: CC BY-NC-SA 3.0 IGO.

[19] Badjatia N Carney N Crocco TJ Fallat ME Hennes HM Jagoda AS. Guidelines for prehospital management of traumatic brain injury 2nd edition. Prehosp Emerg Care. (2008) 12(Suppl.1):S1–52. 10.1080/10903120701732052 18203044

[20] Wooldridge G Hansmann A Aziz O O'Brien N. Survey of resources available to implement severe pediatrictraumatic brain injury management guidelines in low and middle-income countries. Child's Nervous Syst. (2020) 36:2647–55. 10.1007/s00381-020-04603-9 32300872

[21] Fink EL von Saint Andre-von Arnim A Kumar R Wilson P Bacha T Aklilu AT. Traumatic brain injury and infectious encephalopathy in children from four resource limited settings in Africa. Pediatr Crit Care Med. (2018) 19:649–57. 10.1097/PCC.0000000000001554 29664874

[22] Kochanek PM Tasker RC Carney N Totten AM Adelson PD Selden NR Guidelines for the management of paediatric severe traumatic brain injury, third edition: update of the brain trauma foundation guidelines. Paediatr Crit Care Med. (2019) 20(Suppl.1):S1–82. 10.1097/PCC.0000000000001735 30829890

[23] Kochanek PM Tasker RC Bell MJ Adelson PD Carney N Vavilala MS. Management of pediatric severe traumatic brain injury: 2019 consensus and guidelines-based algorithm for first and second tier therapies. Pediatr Crit Care Med. (2019) 20:269–79. 10.1097/PCC.0000000000001737 30830015

[24] Pedersen SH Liljia-Cyron A Astrand R Juhler M. Monitoring and measurement of intracranial pressure in pediatric head trauma. Front Neurol. (2020) 10:1376. 10.3389/fneur.2019.01376 32010042 PMC6973131

[25] Larsen GY Schober M Fabio A Wisniewki SR Grant M Shafi N Structure, process, and culture differences of pediatric trauma centres participating in an international comparative effectiveness study of children with severe traumatic brain injury. Neurocrit Care. (2016) 24:353–60. 10.1007/s12028-015-0218-6 26627225 PMC4884520

[26] Figaji AA Fieggen AG Peter JC. Early decompressive craniotomy in children with severe traumatic brain injury. Childs Nerv Syst. (2003) 19:666–73. 10.1007/s00381-003-0804-3 12908115

[27] Alali AS Temkin N Barber J Pridgeon J Chaddock K Dikmen S. A clinical decision rule to predict intracranial hypertension in severe traumatic brain injury. J Neurosurg. (2018) 131:612–19. 10.3171/2018.4.JNS173166 30265194 PMC6586526

[28] Hendrickson P Pridgeon J Temkin NR Videtta V Petroni G Lujian S. Development of a severe traumatic brain injury consensus-based treatment protocol conference in Latin America. World Neurosurg. (2018) 110:e952–7. 10.1016/j.wneu.2017.11.142 29203307 PMC6214355

[29] Le Roux P Menon DK Citerio G Vespa P Kay Bader M Brophy GM Consensus summary statement of the international multidisciplinary consensus conference on multimodality monitoring in neurocritical care a statement for healthcare professionals from the neurocritical care society and the european society of intensive care medicine. Neurocrit Care. (2014) 21(Suppl.2):S1–26. 10.1007/s00134-014-3369-6 25208678 PMC10596301

[30] Chesnut RM Temkin N Carney N Dikmen S Rondina C Videtta W. Trial of intracranial-pressure monitoring in traumatic brain injury. N Engl J Med. (2012) 367:2471–81. 10.1056/NEJMoa1207363 23234472 PMC3565432

[31] Bennett TD Riva-Cambrin J Keenan HT Korgenski EK Bratton SL. Variation in intracranial pressure monitoring and outcomes in pediatric traumatic brain injury. Arch Ped Adolesc Med. (2012) 166:641–7. 10.1001/archpediatrics.2012.322 22751878 PMC4547349

[32] Bennett TD DeWitt PE Greene TH Srivastava R Riva-Cambrin J Nance ML. Functional outcome after intracranial pressure monitoring for children with severe traumatic brain injury. JAMA Pediatrics. (2017) 171:965–71. 10.1001/jamapediatrics.2017.2127 28846763 PMC5710627

[33] Kukreti V Mohseni-Bod H Drake J. Management of raised intracranial pressure in children with traumatic brain injury. J Ped Neurosci. (2014) 9:207–15. 10.4103/1817-1745.147572 25624921 PMC4302538

[34] Schrieff-Elson LE Thomas KGF Rohlwink UK Figaji AA. Low brain oxygenation differences in neuropsychological outcomes following severe pediatric TBI. Childs Nerv Syst. (2015) 31:2257–68. 10.1007/s00381-015-2892-2 26337700

[35] Ommaya AK Goldsmith W Thibault L. Biomechanics and neuropathology of adult and paediatric head injury. Br J Neurosurg. (2002) 16:220–42. 10.1080/02688690220148824 12201393

[36] Kochanek PM Jha RM Clark RSB. “Take a number”- precision monitoring directs precision therapy. Neurocrit Care. (2020) 32:683–6. 10.1007/s12028-020-00941-3 32141034 PMC7275915

[37] Donnelly JE Young AMH Brady K. Autoregulation in paediatric TBI—current evidence and implications for treatment. Child's Nervous Syst. (2017) 33:1735–44. 10.1007/s00381-017-3523-x 29149389

[38] Ducharme-Crevier L. Cerebrovascular pressure reactivity in children with TBI. Pediatr Neurol Briefs. (2015) 29:77. 10.15844/pedneurbriefs-29-10-4 26933535 PMC4747139

[39] Lewis PM Czosnyka M Carter BJ Rosenfeld JV Paul E Singhal N. Cerebrovascular pressure reactivity in children with traumatic brain injury. Pediatr Crit Care Med. (2015) 16:739–49. 10.1097/PCC.0000000000000471 26132743

[40] Horvat CM Kochanek PM. Big data not yet big enough to determine the influence of intracranial pressure monitoring on outcome in children with severe traumatic brain injury. JAMA Pediatr. (2017) 171:942–3. 10.1001/jamapediatrics.2017.2390 28846750

[41] Kochanek PM Clark RSB Ruppel RA Adelson PD Bell MJ Whalen MJ. Biochemical, cellular and molecular mechanisms in the evolution of secondary damage after severe traumatic brain injury in infants and children: lessons learned from the bedside. Pediatr Crit Care Med. (2000) 1:4–19. 10.1097/00130478-200007000-00003 12813280

[42] Simon DW McGeachy M Bayir H Clark RSB Loane DJ Kochanek PM The far-reaching scope of neuroinflammation after traumatic brain injury. Nat Rev Neurol. (2017) 13:171–91. 10.1038/nrneurol.2017.13 28186177 PMC5675525

[43] Kochanek PM Jackson TC Jha RM Clark RSB Okonkwo DO Bayir H. Paths to successful translation of new therapies for severe TBI in the golden age of traumatic brain injury research: a pittsburgh vision. J Neurotrauma. (2019) 37:2353–71. 10.1089/neu.2018.6203 30520681 PMC7698994

[44] Adamides AA Cooper DJ Rosenfeldt FL Bailey MJ Pratt N Tippett N Focal cerebral oxygenation and neurological outcome with or without brain tissue oxygen-guided therapy in patients with traumatic brain injury. Acta Neurochirurgica. (2009) 151:1399–409. 10.1007/s00701-009-0398-y 19727549

[45] Figaji AA Zwane E Fieggen AG Argent AC Le Roux PD Peter JC. The effect of increased inspired fraction of oxygen on brain tissue oxygen tension in children with severe traumatic brain injury. Neurocritical Care. (2010) 12:430–37. 10.1007/s12028-010-9344-3 20232264

[46] Figaji AA Zwane E Thompson C Fieggen AG Argent AC Le Roux PD Brain tissue oxygen tension monitoring in pediatric severe traumatic brain injury: part 2: relationship with clinical, physiological, and treatment factors. Child's Nervous Syst. (2009) 25:1335–43. 10.1007/s00381-009-0821-y 19214533

[47] Figaji AA. Practical aspects of bedside cerebral hemodynamics monitoring in pediatric TBI. Child's Nervous Syst. (2010) 26:431–39. 10.1007/s00381-009-1036-y 19937247

[48] Al-Mufti F Lander M Smith B Morris NA Nuoman R Gupta R. Multimodality monitoring in neurocritical care: decision-making utilizing direct and indirect surrogate markers. J Intensive Care Med. (2019) 34:449–63. 10.1177/0885066618788022 30205730

[49] Young AMH Guilfoyle MR Donnelly J Smielewski P Agarwal S Czosnyka M. Multimodality neuromonitoring in severe pediatric traumatic brain injury. Pediatric Res. (2018) 83:41–49. 10.1038/pr.2017.215 29084196

[50] Burstein B Upton JEM Terra HF Neuman MI. Use of CT for head trauma: 2007-2015. Paediatrics. (2018) 142:e20180814. 10.1542/peds.2018-0814 30181120

[51] Currie S Saleem N Straiton JA Macmullen-Price J Warren DJ Craven IJ. Imaging assessment of traumatic brain injury. Postgrad Med J. (2016) 92:41–50. 10.1136/postgradmedj-2014-133211 26621823

[52] Pearce MS Salotti JA Little MP McHugh K Lee C Kim KP. Radiation exposure from CT scans in childhood and subsequent risk of leukaemia and brain tumours: a retrospective cohort study. Lancet. (2012) 380:499–505. 10.1016/S0140-6736(12)60815-0 22681860 PMC3418594

[53] Miglioretti DL Johnson E Williams A Greenlee RT Weimann S Solberg LI. The use of computed tomography in pediatrics and the associated radiation exposure and estimated cancer risk. JAMA Pediatr. (2013) 167:700–7. 10.1001/jamapediatrics.2013.311 23754213 PMC3936795

[54] Da Dalt L Parri N Amigoni A Nocerino A Selmni F Manara R. Italian guidelines on the assessment and management of pediatric head injury in the emergency department. Ital J Ped. (2018) 44:7. 10.1186/s13052-017-0442-0 29334996 PMC5769508

[55] Babl FE Borland ML Phillips N Kochar A Dalton S McCaskill M. Accuracy of PECARN, CATCH, and CHALICE head injury decision rules in children: a prospective cohort study. Lancet. (2017) 389:2393–402. 10.1016/S0140-6736(17)30555-X 28410792

[56] Ferrazzano PA Rosario BL Wisniewski SR Shafi NI Siefkes HM Miles DK. Use of magnetic resonance imaging in severe pediatric traumatic brain injury: assessment of current practice. J Neurosurg Ped. (2019) 23:471–9. 10.3171/2018.10.PEDS18374 30738383 PMC6687576

[57] Lindberg DM Stence NV Grubenhoff JA Lewis T Mirsky DM Miller AI. Feasibility and accuracy of fast MRI versus CT for traumatic brain injury in young children. Pediatrics. (2019) 144:e20190419. 10.1542/peds.2019-0419 31533974

[58] Parri N Crosby BJ Mills L Soucy Z Musolino AM Da Dalt L. Point-of-care ultrasound for the diagnosis of skull fractures in children younger than two years of age. J Pediatrics. (2018) 196:230–6. 10.1016/j.jpeds.2017.12.057 29499992

[59] Raffiz M Abdullah JM. Optic nerve sheath diameter measurement: a means to detecting raised ICP in adult traumatic and non-traumatic neurosurgical patients. Am J Emerg Med. (2017) 35:150–3. 10.1016/j.ajem.2016.09.044 27852525

[60] Herman ST Abend NS Bleck TP Chapman KE Drislane FW Emerson RG. Critical care continuous EEG task force of the American clinical neurophysiology society: consensus statement on continuous EEG in critically ill adults and children, part I: indications. J Clin Neurophysiol. (2015) 32:87–95. 10.1097/WNP.0000000000000166 25626778 PMC4435533

[61] Kurz JE Poloyac SM Abend NS Fabio A Bell MJ Wainwright MS. Variation in anticonvulsant selection and electroencephalographic monitoring following severe traumatic brain injury in children-understanding resource availability in sites participating in a comparative effectiveness study. Pediatr Crit Care Med. (2016) 17:649–57. 10.1097/PCC.0000000000000765 27243415 PMC5189641

[62] Ostahowski PJ Kannan N Wainwright MS Qiu Q Mink RB Groner JI. Variation in seizure prophylaxis in severe pediatric traumatic brain injury. J Neurosurg Pediatr. (2016) 18:499–506. 10.3171/2016.4.PEDS1698 27258588

[63] Carney N Totten AM O'Reilly C Ullman JS Hawryluk GWJ Bell MJ. Guidelines for the management of severe traumatic brain injury, fourth edition. Neurosurgery. (2017) 80:6–15. 10.1227/NEU.0000000000001432 27654000

[64] Hutchinson PJ Kolias AG Timofeev IS Corteen EA Czosnyka M Timothy J. Trial of decompressive craniectomy for traumatic intracranial hypertension. N Engl J Med. (2016) 375:1119–30. 10.1056/NEJMoa1605215 27602507

[65] Young AMH Kolias AG Hutchinson PJ. Decompressive craniectomy for traumatic intracranial hypertension: application in children. Childs Nevr Syst. (2017) 33:1745–50. 10.1007/s00381-017-3534-7 29149391 PMC5587789

[66] Ballestero MFM Furlanetti LL Augusto LP Chaves PHC Santos MV de Oliveira RS. Decompressive craniectomy for severe traumatic brain injury in children: analysis of long-term neuropsychological impairment and review of the literature. Childs Nerv Syst. (2019) 35:1507–15. 10.1007/s00381-019-04274-1 31264065

[67] Manfiotto M Mottolese C Szathmari A Beuriat P Klein O Vinchon M. Decompressive craniectomy and CSF disorders in children. Childs Nerv Syst. (2017) 33:1751–7. 10.1007/s00381-017-3542-7 29149390

[68] Kolias A Viaroli E Rubiano AM Adams H Khan T Gupta D. The current status of decompressive craniectomy in traumatic brain injury. Curr Trauma Rep. (2018) 4:326–32. 10.1007/s40719-018-0147-x 30473990 PMC6244550

[69] Hutchinson PJ Kolias AG Tajsic T Adeleye A Akliu AT. Consensus statement from the international consensus meeting on the role of decompressive craniectomy in the management of traumatic brain injury. Acta Neurochirurgica. (2019) 161:1261–74. 10.1007/s00701-019-03936-y 31134383 PMC6581926

[70] Bonow RH Oron AP Hanak BW Browd SR Chestnut RM. Post-traumatic hydrocephalus in children: a retrospective study in 42 pediatric hospitals using the pediatric health information system. Neurosurgery. (2018) 83:732–9. 10.1093/neuros/nyx470 29029289

[71] Frassanito P Massimi L Caldarelli M Tamburrini G Di Rocco C. Complications of delayed cranial repair after decompressive craniectomy in children less than 1 year old. Acta Neurochir. (2012) 154:927–33. 10.1007/s00701-011-1253-5 22198327

[72] Piedra MP Thompson EM Selden NR Ragel BT Guillame DJ. Optimal timing of autologous cranioplasty after decompressive craniectomy in children. J Neurosurg Pediatr. (2012) 10:268–72. 10.3171/2012.6.PEDS1268 22861195

[73] Grant GA Jolley M Ellenbogen RG Roberts TS Gruss JR Loeser JD. Failure of autologous bone-assisted cranioplasty following decompressive craniectomy in children adolescents. J Neurosurg. (2004) 100(2 Suppl):163–8. 10.3171/ped.2004.100.2.0163 14758944

[74] Martin KD Franz B Kirsch M Polanski W von der Hagen M Schackert G. Autologous bone flap cranioplasty following decompressive craniectomy is combined with a high complication rate in pediatric traumatic brain injury patients. Acta Neurochir. (2014) 156:813–24. 10.1007/s00701-014-2021-0 24532225

[75] Bowers CA Riva-Cambrin J Hertzler DA Walker ML. Risk factors rates of bone flap resorption in pediatric patients after decompressive craniectomy for traumatic brain injury. J Neurosurg Pediatr. (2013) 11:526–32. 10.3171/2013.1.PEDS12483 23473303

[76] Frassanito P Massimi L Caldarelli M Tamburrini G Di Rocco C Letter to the editor: bone flap resorption in infants. J Neurosurg Pediatr. (2014) 13:243–4. 10.3171/2013.6.PEDS13312 24313656

[77] Klieverik VM Miller KJ Singhal A Han KS Woederman PA. Cranioplasty after craniectomy in pediatric patients-a systematic review. Childs Nerv Syst. (2019) 35:1481–90. 10.1007/s00381-018-4025-1 30610476

[78] Frassanito P Tamburrini G Massimi L Peraio S Caldarelli M Di Rocco C. Problems of reconstructive cranioplasty after traumatic brain injury in children. Childs Nerv Syst. (2017) 33:1759–68. 10.1007/s00381-017-3541-8 29149388

[79] Frassanito P Tamburrini G Massimi L Di Rocco C Nataloni A Fabbri G. Post-marketing surveillance of custom bone service implanted in children under 7 years old. Acta Neurochir. (2015) 157:115–21. 10.1007/s00701-014-2254-y 25326712

[80] Spennato P Canella V Aliberti F Russo C Ruggiero C Nataloni A. Hydroxyapatite ceramic implants for cranioplasty in children: a retrospective evaluation of clinical outcome and osteointegration. Childs Nerv Syst. (2020) 36:551–8. 10.1007/s00381-019-04423-6 31786632

[81] Emami P Czorlich P Fritzsche FS Westphal M Rueger JM Lefering R. Impact of Glasgow coma scale score and pupil parameters on mortality rate and outcome in pediatric and adult severe traumatic brain injury: a retrospective, multicenter cohort study. J Neurosurg. (2017) 126:760–7. 10.3171/2016.1.JNS152385 27035177

[82] Murphy S Thomas NJ Gertz SJ Beca J Luther JF Bell MJ. Tripartite stratification of the Glasgow coma scale in children with severe traumatic brain injury and mortality: an analysis from a multi-center comparative effectiveness study. J Neurotrauma. (2017) 34:2220–9. 10.1089/neu.2016.4793 28052716 PMC5510706

[83] Fulkerson DH White IK Rees JM Baumanis MM Smith JL Ackerman LL. Analysis of long-term (median 10.5 years) outcomes in children presenting with traumatic brain injury and an initial Glasgow coma scale score of 3 or 4. J Neurosurg Pediatric. (2015) 16:410–19. 10.3171/2015.3.PEDS14679 26140392

[84] Forsyth R Would you rather have your brain injury at five or twenty-five? Dev Med Child Neurol. (2014) 56:297 10.1111/dmcn.12397 24506580

[85] Sarnaik A Ferguson NM O'Meara AMI Agrawal S Deep A Buttram S. Age and mortality in pediatric severe traumatic brain injury: results from an international study. Neurocrit Care. (2018) 28:302–13. 10.1007/s12028-017-0480-x 29476389 PMC10655613

[86] Königs M Heij HA van der Sluijs JA Vermulen RJ Goslings JC Luitse JSK. Pediatric traumatic brain injury and attention deficit. Pediatrics. (2015) 136:534–41. 10.1542/peds.2015-0437 26240208

[87] Babikian T Merkeley T Savage RC Giza CC Levin H. Chronic aspects of pediatric traumatic brain injury: review of the literature. J Neurot. (2015) 32:1849–60. 10.1089/neu.2015.3971 26414654

[88] McCauley SR Wilde EA Anderson VA Bedell G Beers SR Campbell TF. Pediatric TBI outcomes workgroup. Recommendations for the use of common outcome measures in pediatric traumatic brain injury research. J Neurotrauma. (2012) 29:678–705. 10.1089/neu.2011.1838 21644810 PMC3289848

[89] Schrieff-Elson LE Steenkamp N Hendricks MI Thomas KGF Rohlwink UK. Local and global challenges in pediatric traumatic brain injury outcome and rehabilitation assessment. Childs Nerv Syst. (2017) 33:1775–84. 10.1007/s00381-017-3527-6 29149382

[90] Maddux AB Cox-Martin M Dichiaro M Bennett TD. The association between the functional status scale and the pediatric functional independence measure in children who survive traumatic brain injury. Pediatr Crit Care Med. (2018) 19:1046–53. 10.1097/PCC.0000000000001710 30119094 PMC6218283

[91] Slater A Shann F Pearson G PIM Study Group. PIM2: a revised version of the paediatric index of mortality. Intensive Care Med. (2003) 29:278–85. 10.1007/s00134-002-1601-2 12541154

[92] Berger RP Adelson PD Richichi R Kochanek PM. Serum biomarkers after traumatic and hypoxemic brain injuries: insight into the biochemical response of the pediatric brain to inflicted brain injury. Dev Neurosci. (2006) 28:327–35. 10.1159/000094158 16943655

[93] Papa L Brophy GM Welch RD Lewis LM Braga CF Tan CN Time course and diagnostic accuracy of glial and neuronal blood biomarkers GFAP and UCH-L1 in a large cohort of trauma patients with and without mild traumatic brain injury. JAMA Neurol. (2016) 73:551–60. 10.1001/jamaneurol.2016.0039 27018834 PMC8805143

[94] Kochanek PM Berger RP Bayir H Wagner AK Jenkins LW Clark RS. Biomarkers of primary and evolving damage in traumatic and ischemic brain injury: diagnosis, prognosis, probing mechanisms, and therapeutic decision making. Curr Opin Crit Care. (2008) 14:135–41. 10.1097/MCC.0b013e3282f57564 18388674

[95] Curtis K Foster K Mitchell R Van C. Models of care delivery for families of critically ill children: an integrative review of international literature. J Pediatr Nurs. (2016) 31:330–41. 10.1016/j.pedn.2015.11.009 26699441

[96] Moore M Robinson G Mink R Hudson K Dotolo D Gooding T. Developing a family-centreed care model for critical care after pediatric traumatic brain injury. Pediatr Crit Care Med. (2015) 16:758–65. 10.1097/PCC.0000000000000494 26135064 PMC4592380

[97] Pozzi M Galbiati S Locatelli F Carnovale C Gentili M Radice S. Severe acquired brain injury Aetiologies, early clinical factors, and rehabilitation outcomes: a retrospective study on pediatric patients in rehabilitation. Brain Injury. (2019) 33:1522–8. 10.1080/02699052.2019.1658128 31446793

[98] Forsyth R Basu A. The promotion of recovery through rehabilitation after acquired brain injury in children. Dev Med Child Neurol. (2015) 57:16–22. 10.1111/dmcn.12575 25200439

[99] Walker TC Kudchadkar SR. Early mobilization in the pediatric intensive care unit. Transl Pediatr. (2018) 7:308–13. 10.21037/tp.2018.09.02 30460183 PMC6212381

[100] Forsyth RJ. Back to the future: rehabilitation of children after brain injury. Arch Dis Child. (2010) 95:554–9. 10.1136/adc.2009.161083 20551199

[101] Anderson V Godfrey C Rosenfeld JV Catroppa C. Predictors of cognitive function and recovery 10 years after traumatic brain injury in young children. Pediatrics. (2012) 129:e254–61. 10.1542/peds.2011-0311 22271691

[102] Beretta E Cesareo A Biffi E Schafer C Galbiati S Strazzer S. Rehabilitation of upper limb in children with acquired brain injury: a preliminary comparative study. J Healthc Eng. (2018) 2018:4208492. 10.1155/2018/4208492 29732047 PMC5872655

[103] Herbsman J Al-Qaqaa Y Corcoran J Daly J Folks T Klein D. Early rehabilitation in the pediatric intensive care unit: a quality improvement project. BMJ Qual Saf. (2016) 25:677. 10.1136/bmjqs-2016-IHIabstracts.232190800

[104] Fink EL Beers SR Houtrow AJ Richichi R Burns C Doughty L. Early protocolized versus usual rehabilitation for pediatric neurocritical care patients: a randomized controlled trial. Pediatr Crit Care Med. (2019) 20:540–50. 10.1097/PCC.0000000000001881 30707210 PMC7112470

[105] Simpson AJ Rivara FP Pham TN Quality of care in pediatric trauma. Int J Crit Illn Inj Sci. (2012) 2:149–55. 10.4103/2229-5151.100893 23181209 PMC3500007

[106] Wainwright MS Grimason M Goldstein J Smith CM Amlie-Lefond C Revivo G. Building a pediatric neurocritical care program: a multidisciplinary approach to clinical practice and education from the intensive care unit to the outpatient clinic. Sem Paed Neurol. (2014) 21:248–54. 10.1016/j.spen.2014.10.006 25727506

[107] Park KB Johnson WD Dempsey RJ. Global neurosurgery: the unmet need. World Neurosurg. (2016) 88:32–35. 10.1016/j.wneu.2015.12.048 26732963

[108] Macerollo A Struhal W Sellner J. Harmonization of European neurology education: the junior doctor's perspective. Neurology. (2013) 81:1626–9. 10.1212/WNL.0b013e3182a9f3ed 24166962

[109] Calero-Martinez SA. Development and assessment of competency-based neurotrauma course curriculum for international neurosurgery residents and neurosurgeons. Neurosurg Focus. (2020) 48:E13. 10.3171/2019.12.FOCUS19850 32114549

[110] Falgiani T Kennedy C Jahnke S Exploration of the barriers and education needs of non-pediatric Hospital Emergency Department Providers in Pediatric Trauma Care. IJCM. (2014) 5:56–62. 10.4236/ijcm.2014.52011

[111] Maas AIR Menon D Lingsma HF Pineda JA Sandel ME Manley GT. Re-orientation of clinical research in traumatic brain injury: report of an international workshop on comparative effectiveness research. J Neurotrauma. (2012) 29:32–46. 10.1089/neu.2010.1599 21545277 PMC3253305

[112] Lu X Zhang L Du H Zhang J Li KK Qu J. Chinese pediatric novel coronavirus study team: SARS-CoV-2 infection in children. N Eng J Med. (2020) 382:1663–5. 10.1056/NEJMc2005073 32187458 PMC7121177

[113] Chen H Guo J Wang C Luo F Yu X Zhang W. Clinical characteristics and intrauterine vertical transmission potential of COVID-19 infection in nine pregnant women: a retrospective review of medical records. Lancet. (2020) 395:809–15. 10.1016/S0140-6736(20)30360-3 32151335 PMC7159281

[114] Nacoti M Ciocca A Giupponi A Brambillasca P Lussana F Pisano M At the epicenter of the covid-19 pandemic and humanitarian crises in Italy: changing perspectives on preparation and mitigation. N Eng J Med. (2020). 10.1186/s12939-020-01162-y

[115] Faustini P Rutstein D From the Global Epicenter of the COVID-19 Pandemic, Insights on Helping Families and Children Cope. Available online at: https://blogs.unicef.org/evidence-for-action/from-the-global-epicenter-of-the-covid-19-pandemic-insights-on-helping-families-and-children-cope/ (accessed December 22, 2020)

[116] Social determinant of health. WHO Called to Return to the Declaration of Alma-Ata. Available online at: https://www.who.int/social_determinants/tools/multimedia/alma_ata/en/ (accessed May 13, 2020).

[117] Coccolini F Catena F Gamberini E Ierardi AM Sartelli M Nacoti M Trauma management during and after COVID-19. JEVTM. (2020) Available online at: https://www.jevtm.com/journal/images/v4n1/jevtm_vol4_iss1_2020.pdf (accessed December 22, 2020).

[118] Livingston EH. Surgery in a time of uncertainty. A need for universal respiratory precautions in the operating room. JAMA. (2020) 323:2254–55. 10.1001/jama.2020.7903 32379271

[119] Ong JSM Tosoni A Kim YJ Kisson N Srinivas M. Coronavirus disease 2019 in critically ill children: a narrative review of the literature. Pediatr Crit Care Med. (2020) 21:662–6. 10.1097/PCC.0000000000002376 32265372 PMC7176259

[120] Turolla A Rossettini G Viceconti A Palese A Geri T. Musculoskeletal physical therapy during the COVID-19 pandemic: is telerehabilitation the answer? Phys Ther. (2020) 100:1260–4. 10.1093/ptj/pzaa093 32386218 PMC7239136

[121] Fazzi E Galli J. New clinical needs and strategies for care in children with neurodisability during COVID-19. Dev Med Child Neurol. (2020) 62:879–80. 10.1111/dmcn.14557 32358977 PMC7267576

[122] Corti C Urgesi C Poggi G Strazzer S Borgatti R Bardoni A. Home-based cognitive training in pediatric patients with acquired brain injury: preliminary results on efficacy of a randomized clinical trial. Sci Rep. (2020) 10:1391. 10.1038/s41598-020-57952-5 31996709 PMC6989528

[123] Camden C Pratte G Fallon F Couture M Berbari J Tousignant M Diversity of practices in telerehabilitation for children with disabilities and effective intervention characteristics: results from a systematic review. Disabil Rehabil. (2019) 12:1–13. 10.1080/09638288.2019.1595750 30978110

[124] Ganapathy K. Telemedicine and neurological practice in the COVID-19 era. Neurol India. (2020) 68:555–9. 10.4103/0028-3886.288994 32643663

[125] Kernic MA Rivara FP Zatzich DF Bell MJ Wainwright MS Groner JI. Triage of children with moderate and severe traumatic brain injury to trauma centres. J Neurotrauma. (2013) 30:1129–36. 10.1089/neu.2012.2716 23343131 PMC3700462

